# Y12C mutation disrupts IMPDH cytoophidia and alters cancer metabolism

**DOI:** 10.1111/febs.70086

**Published:** 2025-04-05

**Authors:** Chia‐Chun Chang, Min Peng, Gerson Dierley Keppeke, Li‐Kuang Tsai, Ziheng Zhang, Li‐Mei Pai, Li‐Ying Sung, Ji‐Long Liu

**Affiliations:** ^1^ Institute of Biotechnology National Taiwan University Taipei Taiwan; ^2^ School of Life Science and Technology ShanghaiTech University China; ^3^ Departamento de Ciencias Biomédicas, Facultad de Medicina Universidad Católica del Norte Coquimbo Chile; ^4^ Department of Biochemistry and Molecular Biology, College of Medicine Chang Gung University Taoyuan Taiwan; ^5^ Liver Research Center Chang Gung Memorial Hospital Taoyuan Taiwan; ^6^ Center for Developmental Biology and Regenerative Medicine National Taiwan University Taipei Taiwan; ^7^ Center for Biotechnology National Taiwan University Taipei Taiwan; ^8^ Agricultural Biotechnology Research Center Academia Sinica Taipei Taiwan; ^9^ Department of Physiology, Anatomy and Genetics University of Oxford UK

**Keywords:** cancer, CRISPR base editor, cytoophidium, glycolysis, IMPDH, pentose phosphate pathway

## Abstract

Guanosine triphosphate (GTP) is a building block for DNA and RNA, and plays a pivotal role in various cellular functions, serving as an energy source, enzyme cofactor and a key component of signal transduction. The activity of the rate‐limiting enzyme in *de novo* GTP synthesis, inosine monophosphate dehydrogenase (IMPDH), is regulated by nucleotide binding. Recent studies have illuminated that IMPDH octamers can assemble into linear polymers, adding another dimension to its enzymatic regulation. This polymerisation reduces IMPDH's sensitivity to the inhibitory effects of GTP binding, thereby augmenting its activity under conditions with elevated GTP levels. Within cells, IMPDH polymers may cluster to form the distinctive structure known as the cytoophidium, which is postulated to reflect the cellular demand for increased GTP concentrations. Nevertheless, the functional significance of IMPDH polymerisation in *in vivo* metabolic regulation remains unclear. In this study, we report the widespread presence of IMPDH cytoophidia in various human cancer tissues. Utilising the ABEmax base editor, we introduced a Y12C point mutation into IMPDH2 across multiple cancer cell lines. This mutation disrupts the polymerisation interface of IMPDH and prevents cytoophidium assembly. In some cancer xenografts, the absence of IMPDH polymers led to a downregulation of *IMPDH*, as well as the glycolytic and pentose phosphate pathways. Furthermore, mutant HeLa‐cell‐derived xenografts were notably smaller than their wild‐type counterparts. Our data suggest that IMPDH polymerisation and cytoophidium assembly could be instrumental in modulating metabolic homeostasis in certain cancers, offering insights into the clinical relevance of IMPDH cytoophidium.

## Graphical abstract

IMPDH, the rate‐limiting enzyme for *de novo* GTP synthesis, can form polymers and cytoophidia to enhance activity by reducing GTP inhibition. In this study, a Y12C mutation in IMPDH2 was introduced via ABEmax base‐editing in human cancer cell lines to disrupt IMPDH polymerisation. In HeLa cell‐derived xenografts, mutant tumours showed downregulated glycolysis, PPP gene expression, and tumour growth, highlighting the role of IMPDH polymerisation in cancer metabolism.

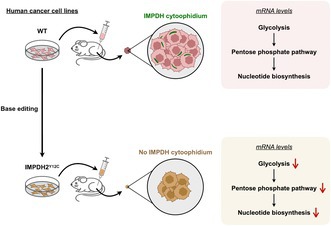

**Abbreviations**
ADP: adenosine diphosphate
AMP: adenosine monophosphate
ATP: adenosine triphosphate
CTP: cytidine triphosphate
DAPI: 4′,6‐diamidino‐2‐phenylindole
DHAP: dihydroxyacetone phosphate
EdU: 5‐ethynyl‐2′‐deoxyuridine
G6P: glucose 6‐phosphate
GDP: guanosine diphosphate
GTP: guanosine triphosphate
IDH: isocitrate dehydrogenase
IMP: inosine monophosphate
IMPDH: inosine monophosphate dehydrogenase
MPA: mycophenolic acid
PGK: phosphoglycerate kinase
PPP: pentose phosphate pathway
PRPP: phosphoribosyl pyrophosphate
PRPS: phosphoribosyl pyrophosphate synthetase
R5P: ribose 5‐phosphate
Ru5P: ribulose 5‐phosphate
S7P: sedoheptulose 7‐phosphate
sgRNA: single guide RNA
UMP: uridine monophosphate
XMP: xanthosine monophosphate

## Introduction

In the cell, GTP serves as the building block of DNA and RNA and participates in various cellular functions, including acting as an energy source, an enzyme cofactor and a component of signal transduction. GTP can be generated through two pathways: the salvage pathway and the *de novo* pathway. In the salvage pathway, guanine nucleotides are produced by recycling guanine. In the *de novo* pathway, the sugar PRPP is converted into IMP and subsequently used by the rate‐limiting enzyme IMPDH to generate XMP, the precursor of guanine nucleotides.

There are two IMPDH isoforms encoded in the human genome. *IMPDH1* is constantly expressed at relatively low levels in most tissues except the retina, whereas *IMPDH2* usually plays the predominant role, especially in fast‐growing tissues [1]. IMPDH activity is regulated by the binding of ADP/ATP and GDP/GTP at multiple binding sites on the CBS domains, altering the interaction between the two IMPDH tetramers in an octamer, which is the active state of IMPDH [2, 3, 4]. While GDP/GTP binding downregulates IMPDH activity by stabilising IMPDH octamers in an inhibited, compressed conformation, ADP/ATP can compete with GDP/GTP binding and stabilise octamers in an active, extended conformation [2, 3, 4]. Biochemical analyses have shown that human IMPDH1/2 activity is significantly suppressed by GDP/GTP at physiological levels [3, 4, 5, 6].

Both IMPDH1 and IMPDH2 octamers can assemble into linear polymers *in vitro* and *in vivo* [3, 7]. The polymerisation of IMPDH desensitises the enzyme to end‐product inhibition, allowing it to maintain activity even under conditions with relatively higher GTP concentrations [3, 4, 6]. Elongation of IMPDH polymers is promoted by the binding of its substrates ATP and IMP, whereas the binding of guanine nucleotides destabilises the polymer [3, 4]. Under certain conditions, IMPDH polymers can further bundle up, forming micron‐scale filaments termed the cytoophidium (cytoophidia for plural) [3, 8, 9].

We have previously demonstrated that the formation of IMPDH cytoophidium is regulated by the abundance of IMPDH polymers/filaments and the molecular crowding status of the cell [9]. These findings also differentiate the factors inducing the assembly of IMPDH filaments and cytoophidia. That is, IMPDH filaments may exist within the cell even when the cytoophidium is not detectable. When the conditions are favourable for IMPDH cytoophidium assembly, both isoforms participate in the same cytoophidium structure [10]. In addition to the potential function of fine‐tuning GTP production, the cytoophidium may also prolong the lifespan of IMPDH proteins [3, 9]. Therefore, polymerisation and cytoophidium assembly of IMPDH have been proposed to provide additional layers of regulation to GTP homeostasis.

IMPDH cytoophidia have been found in various tissues in mammalian models, including humans [8, 11, 12, 13, 14, 15, 16, 17, 18]. In general, cells with IMPDH cytoophidia are relatively more active in GTP‐dependent signalling pathways, such as pancreatic islet cells and photoreceptor cells in the retina, or are highly proliferative, such as activated lymphocytes, pluripotent stem cells and cancer cells [13, 14, 15, 16, 18]. It is reasonable to suspect that IMPDH polymerisation helps maintain higher GTP levels in these cells to meet increased demand. However, a long‐standing question is whether IMPDH polymerisation has physiological significance *in vivo*.

Disruption of the IMPDH polymer interface with a single point mutation, Y12A, has been proven to abrogate IMPDH polymerisation without disturbing its catalytic machinery [3, 7]. These findings provide a promising target for investigating the importance of IMPDH polymers *in vivo*. In this study, we demonstrate a novel strategy for specifically targeting IMPDH polymerisation in the cell using an ABEmax‐based genome‐editing approach. We also show that IMPDH cytoophidia can be found in many human cancers, including multiple types of human cervical cancers. By using this strategy, we introduced a point mutation at residue 12 of IMPDH2 in various cancer cell lines, effectively preventing IMPDH polymerisation and cytoophidium assembly. Although no growth defect was observed in cultured cells, tumour grafts derived from IMPDH2^Y12C^ mutant HeLa cells were significantly smaller than wild‐type grafts. Metabolomic analysis and quantitative PCR reveal that the glycolytic pathway and pentose phosphate pathway (PPP) in mutant tumours were significantly downregulated, suggesting that IMPDH polymerisation broadly impacts the metabolic status of the cell. Similar effects were also found in uterine cancer HEC‐1‐A cells. Collectively, our findings not only demonstrate an effective approach to precisely disrupt IMPDH polymerisation but also suggest the importance of IMPDH polymerisation and cytoophidium assembly in maintaining the metabolic homeostasis of certain cancers.

## Results

### Disrupting IMPDH polymerisation by introducing point mutation via genomic base‐editing

Disruption of IMPDH octamer‐octamer interface with a point mutation at Y12 offers a promising strategy for investigating the functions of IMPDH polymerisation, as this mutation does not affect the catalytic mechanism of IMPDH [3, 7]. Although exogenous expression of the Y12A mutant IMPDH1 or IMPDH2 has been shown to effectively impair cytoophidium assembly in the cell, unexpected effects of *IMPDH* overexpression may perturb the analysis [17, 19]. Therefore, we aimed to introduce the Y12 mutation directly into endogenous IMPDH sequences through genome editing.

**Fig. 1:**
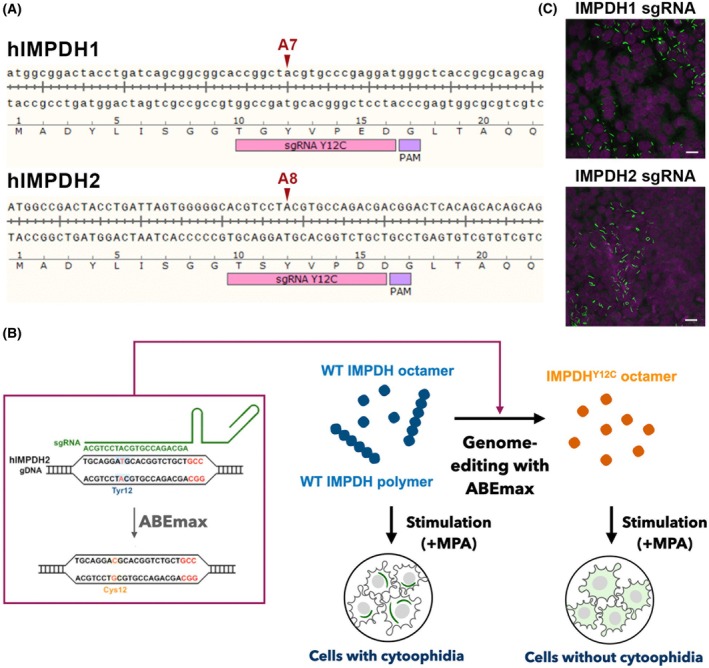
Introducing the Y12C point mutation into IMPDH1 and IMPDH2 in cultured HEK 293T cells using the ABEmax base editor. (A) Genomic sequences of human IMPDH1 and IMPDH2 at the Y12 regions and the corresponding sgRNA sequences. The target adenine (A) residues at positions A7 and A8, which are the intended base‐editing sites, are indicated by arrows. (B) Diagram showing the strategy for establishing no‐cytoophidium cell lines. (C) Immunofluorescence staining of IMPDH (green) and DAPI (magenta) in HEK 293T cells co‐transfected with ABEmax‐T2A‐mCherry and sgRNA constructs targeting IMPDH1 or IMPDH2. Cells are treated with MPA (100 μm) for 1 h before fixation. Scale bars are 20 μm. Images are representative of *n* = 3 independent experiments performed in triplicate.

The ABEmax is a novel dCas9‐based tool designed for targeted A‐to‐G base‐editing in the mammalian genome, with an extremely low off‐target rate [20]. Optimal performance of ABEmax is achieved when the target ‘A’ is located between positions 4 and 8 of the guide RNA [21]. We found that the codon for Y12 (UAC) is conserved in both human and mouse IMPDH1 and IMPDH2, and is located at positions A7 and A8, respectively. In addition, no additional ‘A’ residues are present between positions 2 and 14 in both *IMPDH1* and *IMPDH2* sequences, making them ideal targets for dCas9‐based ABEmax base editing (Fig. 1A,B).

To evaluate the base‐editing efficiency, we first tested *IMPDH1* and *IMPDH2* in HEK 293T cells. A plasmid encoding ABEmax‐T2A‐mCherry and a plasmid encoding sgRNA and puromycin resistance gene were co‐transfected into cells. After a single transfection and selection round, a portion of cells transfected with sgRNAs targeting either isoform exhibited no IMPDH cytoophidium under treatment with an IMPDH inhibitor, mycophenolic acid (MPA), which usually induces IMPDH cytoophidium assembly in nearly all cells (Fig. 1C). These results show that our base‐editing strategy effectively prevents cytoophidium assembly in cells when either IMPDH isoform is mutated.

We have previously demonstrated that a small deletion (6 to 8 amino acids) in the CBS domain of IMPDH2 also prevents cytoophidium assembly in HeLa cells. These mutant cells exhibited no growth defects under normal conditions unless *IMPDH2* was knocked down [10], indicating that the formation of IMPDH polymers or cytoophidia becomes evident under conditions with inadequate nutrients. This is supported by the fact that cytoophidium formation can be induced by nutrient deprivation [22, 23]. To further investigate the physiological significance of IMPDH polymerisation, we aim to examine the IMPDH2^Y12C^ mutant cells under both *in vitro* and *in vivo* conditions. To achieve this, cancer cell lines will serve as our model for *in vitro* manipulation and provide suitable samples for *in vivo* studies after cell transplantation into host mice.

### IMPDH forms cytoophidia in various human cancers

IMPDH cytoophidium formation has been linked to rapid cell proliferation and an active PI3K/AKT/mTOR pathway, which are well‐known characteristics of many cancers [10, 12, 18]. In fact, the abundance of IMPDH cytoophidium can be applied to differentiate acral lentiginous melanomas from melanocytic nevi [15]. In order to further investigate the prevalence of IMPDH cytoophidia in human cancers, we performed an immunofluorescence screening on a human multiple organ tumour microarray.

**Fig. 2:**
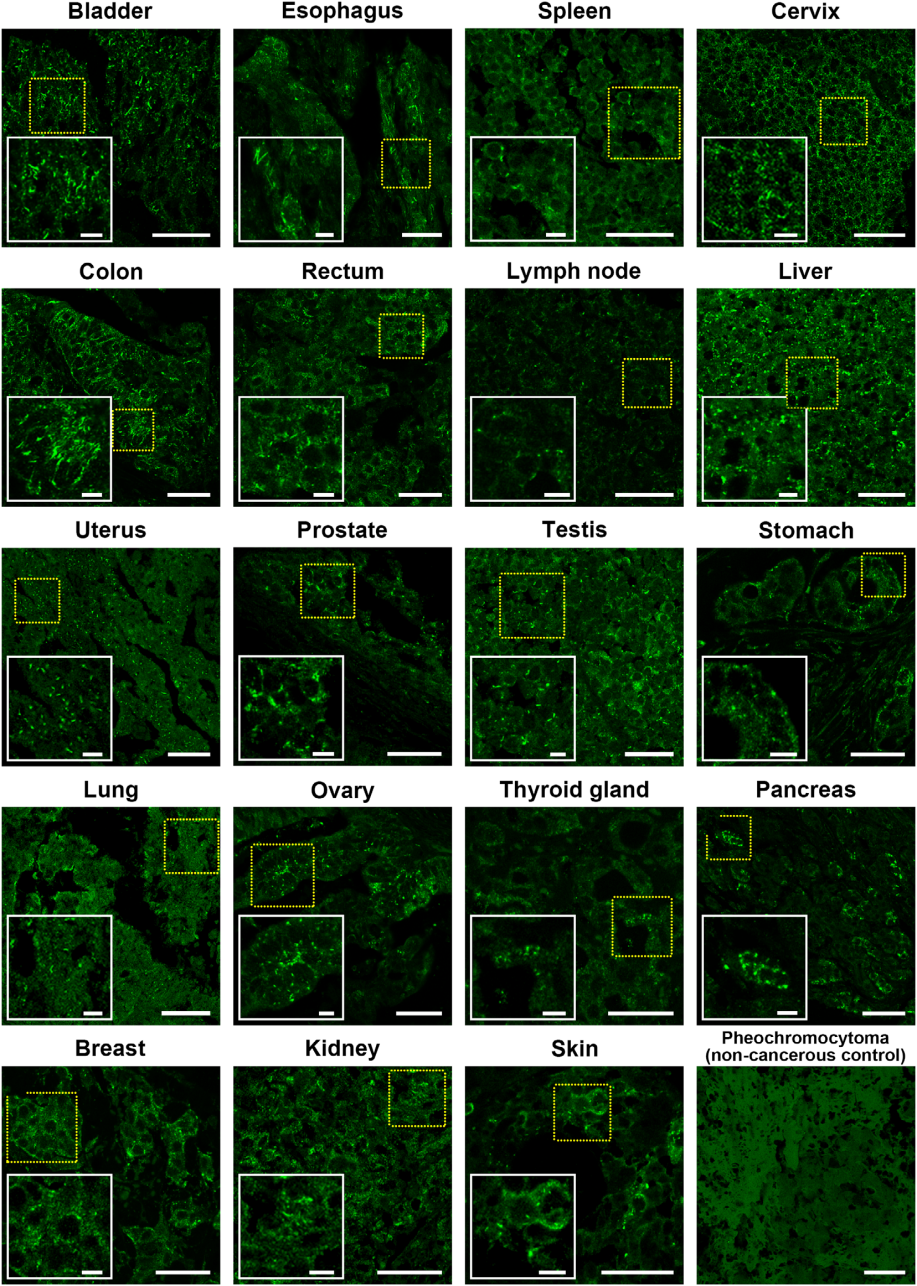
IMPDH forms cytoophidia in various human cancers. Representative images of IMPDH cytoophidium formation in different cancerous tissues, alongside a non‐cancerous pheochromocytoma (no cytoophidium) as a control. All sections are stained with an anti‐IMPDH antibody (green). For each cancer type, all tissue samples are analysed, with three images taken per sample; one representative image is presented. Yellow dashed boxes indicate magnified areas in each image. Scale bars are 50 and 10 μm (insets).

The array included 192 samples from various cancer types across 19 different organs. In more than 60% of bladder, oesophageal, spleen, cervical, colon and lymph node cancer sections, IMPDH cytoophidia could be detected in most regions of the samples. In other cancer types, IMPDH cytoophidia are also present in a smaller subset of samples (Fig. 2 and Table 1). The morphology of cytoophidia varies among tissue types. For instance, cytoophidia in lymphoma and liver cancers mostly appear as dots, while long filaments are more frequently observed in bladder, cervical, and colon cancers (Fig. 2). These morphological differences might result from varying *IMPDH* expression levels, crowding conditions, and the interior structure of the cell. Our previous research has demonstrated that cytoophidium structures are flexible and their shapes are interchangeable [24]. Although small biopsy sections might not fully represent the overall state of the tumours, our results indicate that IMPDH cytoophidium assembly is widespread in various human cancers.

**Table 1:** Expression of cytoophidium in different cancer types on human tumour tissue array.

Cancer type	Total	No. (%) of samples with IMPDH cytoophidium
Bladder	8	8 (100%)
Oesophagus	8	7 (87.5%)
Spleen	8	7 (87.5%)
Cervix	8	6 (75%)
Colon	8	5 (62.5%)
Lymph node	8	5 (62.5%)
Liver	8	4 (50%)
Uterus	8	4 (50%)
Prostate	8	4 (50%)
Testis	8	4 (50%)
Stomach	8	3 (37.5%)
Lung	8	3 (37.5%)
Ovary	16	6 (37.5%)
Thyroid gland	8	2 (25%)
Pancreas	8	1 (12.5%)
Cerebrum	8	1 (12.5%)
Breast	16	2 (12.5%)
Kidney	8	1 (12.5%)
Skin	22	2 (9.1%)

### IMPDH polymerisation and/or cytoophidium formation are required for the maintenance of HeLa cell‐derived tumour growth

**Fig. 3:**
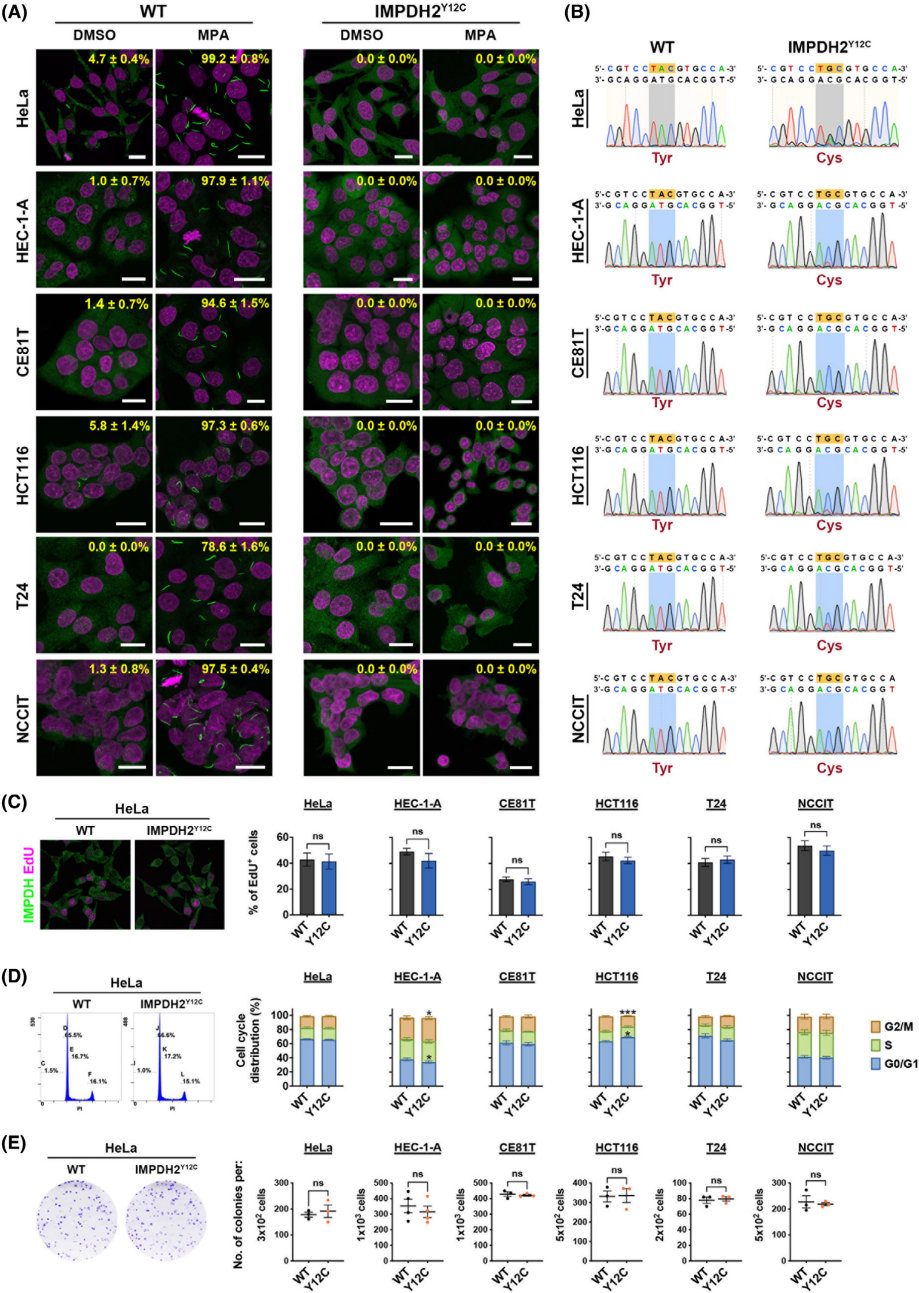
Establishment and *in vitro* characterisation of IMPDH2^Y12C^ mutant human cancer cell lines. (A) Immunofluorescence of wild‐type (WT) and IMPDH2^Y12C^ mutant HeLa, HEC‐1‐A, CE81T, HCT116, T24, and NCCIT cell lines. Cells are treated with 100 μm DMSO or MPA for 1 h before fixation. IMPDH is shown in green, and DAPI is in magenta. Scale bars are 20 μm. Percentages ± SEM in each image indicate the proportion of cells with visible IMPDH cytoophidia. (B) Confirmation of successful genome editing in each mutant cell line by Sanger sequencing of the targeted genomic regions. The edited nucleotide sequences are highlighted in orange, and the corresponding regions in the sequencing chromatograms are shaded in grey/blue. (C) Immunofluorescence of IMPDH (green) and EdU (magenta) in wild‐type and mutant HeLa cells, along with quantitative data showing the proportions of EdU‐positive cells in all wild‐type and mutant cell lines. Scale bars are 20 μm. (D) Flow cytometry analysis of DNA content in wild‐type and mutant HeLa cells, with bar graphs showing the quantitative results of cell proportions based on DNA content in different cell lines. (E) Clonogenic assay results for all wild‐type and mutant cell lines, including representative images of wild‐type and mutant HeLa cell colonies and the quantitative data on the number of colonies derived from all wild‐type and mutant cells. All images are representative of *n* = 3 independent experiments performed in triplicate, and quantification data are obtained from the same experiments. Statistical significance in (C–E) is evaluated using Student's *t*‐test, with error bars representing SEM (*P < 0.05, ***P < 0.001).

Based on the results from the human cancer tissue microarray, we selected six cancer types as candidate models due to their higher tendency to form long cytoophidium filaments. The corresponding cell lines include T24 cells (bladder cancer), CE81T cells (oesophageal cancer), HCT116 cells (colon cancer), HEC‐1‐A cells (uterine cancer), NCCIT cells (testicular cancer) and HeLa cells (cervical cancer). We then transfected these cell lines with the ABEmax/sgRNA construct to target the endogenous *IMPDH2* sequence and established IMPDH2^Y12C^ mutant cell lines (Fig. 3A). All lines were not derived from single‐cell colonies and therefore retained genetic heterogeneity. Sequencing of the PCR products from genomic DNA of each mutant cell line confirmed that not all alleles were edited (Fig. 3B). However, immunofluorescence on MPA‐treated cells shows successful disruption of IMPDH cytoophidium assembly in nearly all cells (Fig. 3A).

We subsequently examined the growth of IMPDH2^Y12C^ mutant cells under culture conditions. The mutant cell lines were cultured in parallel with wild‐type cells, and their proliferation was evaluated by EdU labelling, flow cytometry, and colony formation assay. Despite the mutation in the endogenous IMPDH2, the mutant cell lines showed no significant growth defects compared to wild‐type cells (Fig. 3C–E). Only the mutant HEC‐1‐A and HCT116 cells had a slight increase in the proportion of G0/G1 cells and a decrease in cells in the G2/M stage (Fig. 3D).

**Fig. 4:**
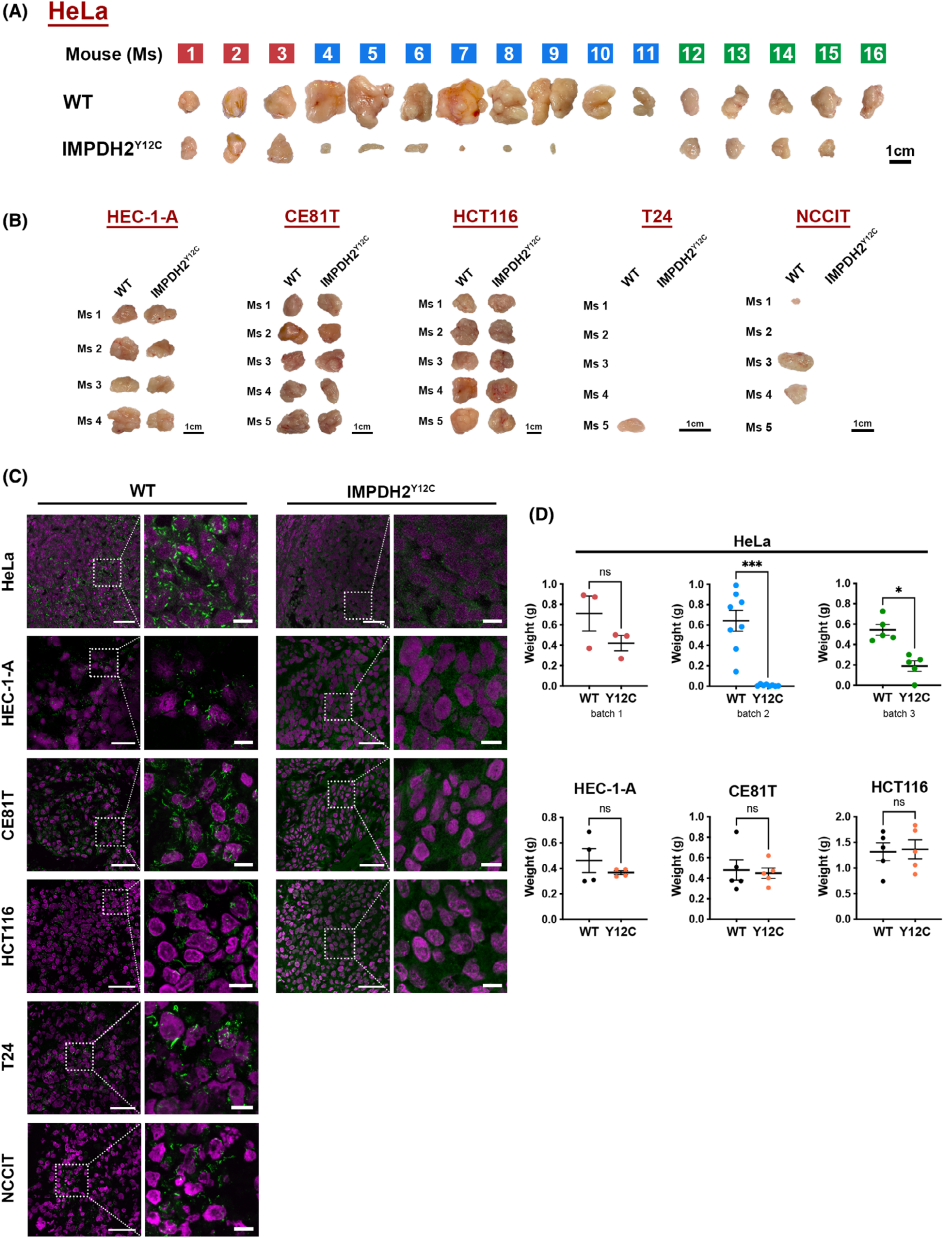
IMPDH2 Y12C point mutation impairs cytoophidium assembly in cancer cell xenografts and disturbs HeLa cell‐derived tumour growth. (A) Images of tumours derived from wild‐type (WT) and IMPDH2^Y12C^ HeLa cells in 16 immune‐deficient mice (numbered). Each host mouse is injected with wild‐type and mutant cells on opposite sides of the thigh muscle. Colour codes indicate different transplantation batches. (B) Images of tumours derived from wild‐type and IMPDH2^Y12C^ HEC‐1‐A, CE81T, HCT116, T24, and NCCIT cells in immune‐deficient mice (numbered). Scale bars are 1 cm. (C) Immunofluorescence of IMPDH (green) and DAPI (magenta) in tumour sections from HeLa, HEC‐1‐A, CE81T, HCT116, T24, and NCCIT cells. Dashed boxes indicate magnified regions in each image. Scale bars are 50 μm in original images and 10 μm in magnified images. (D) Comparison of the weights of wild‐type and mutant xenografts derived from HeLa cells (displayed in three separate batches) and three other cell types. Statistical significance is evaluated using Student's *t*‐test, with error bars representing SEM (*P < 0.05, ***P < 0.001).

We then transplanted all cell lines into immune‐deficient mice and collected the tumour grafts after 5 weeks (Fig. 4A,B). While wild‐type HeLa, HEC‐1‐A, CE81T, and HCT116 cells gave rise to tumour xenografts in all mice, T24 and NCCIT cells failed to form tumours in some hosts. In the wild‐type tumour tissues derived from all six cancer cell lines, IMPDH cytoophidia could be found in most areas of sections, whereas no cytoophidium was observed in the mutant tumours (Fig. 4C). Among the six cancer cell lines, HeLa cell‐derived IMPDH2^Y12C^ mutant tumour grafts showed significantly smaller than wild‐type tumours. In contrast, IMPDH2^Y12C^ mutant T24 and NCCIT cells did not form any tumours in the host mice (Fig. 4A,D). Due to the inability of T24 and NCCIT cells to robustly form tumours in host mice, we decided not to proceed with further analyses for these two cell lines. We performed three replicates in which a total of 16 mice were transplanted with HeLa cells. Although tumour sizes varied between batches, the mutant tumours are significantly smaller than their wild‐type counterparts in all three batches (Fig. 4A,D). These distinct results among these cell lines might reflect their different metabolic preferences and/or their ability to adapt to the loss of IMPDH polymers.

### IMPDH protein levels are not significantly decreased in most IMPDH2^Y12C^ mutant xenografts

**Fig. 5:**
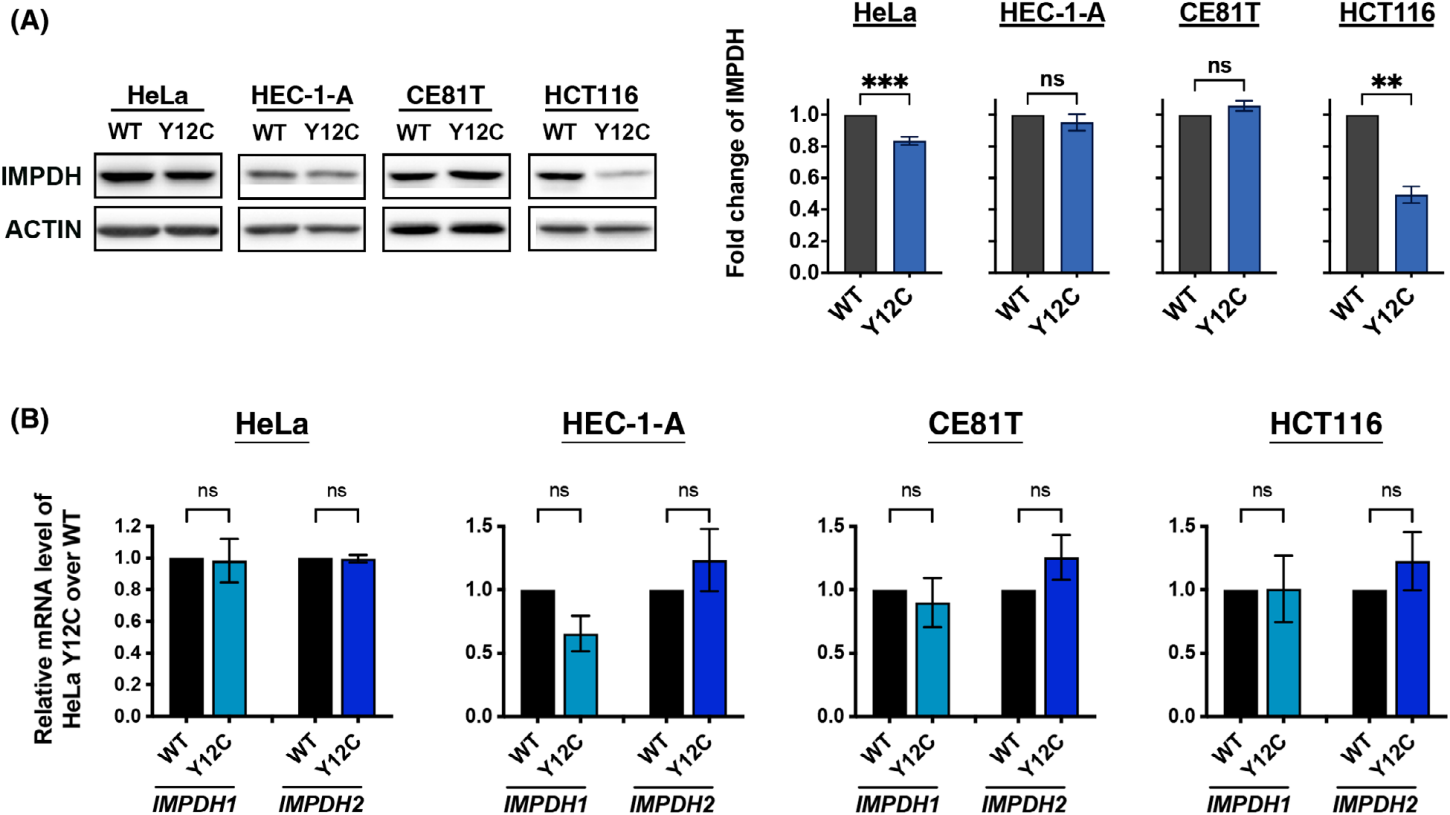
IMPDH2 Y12C point mutation lowers IMPDH levels in some cell types *in vitro*. (A) Western blot and quantification of IMPDH (normalised to ACTIN) in cultured HeLa, HEC‐1‐A, CE81T, and HCT116 cells. Lower IMPDH levels are observed in mutant HeLa and HCT116 cells. (B) Real‐time PCR analysis of IMPDH1 and IMPDH2 mRNA levels in cultured cells. No significant difference in expression levels is observed in any of the mutant cell lines. Images are representative of *n* = 3 independent experiments performed in triplicate, and quantification data are obtained from the same experiments. Statistical significance is evaluated using Student's *t*‐test, with error bars representing SEM (**P < 0.01, ***P < 0.001).

**Fig. 6:**
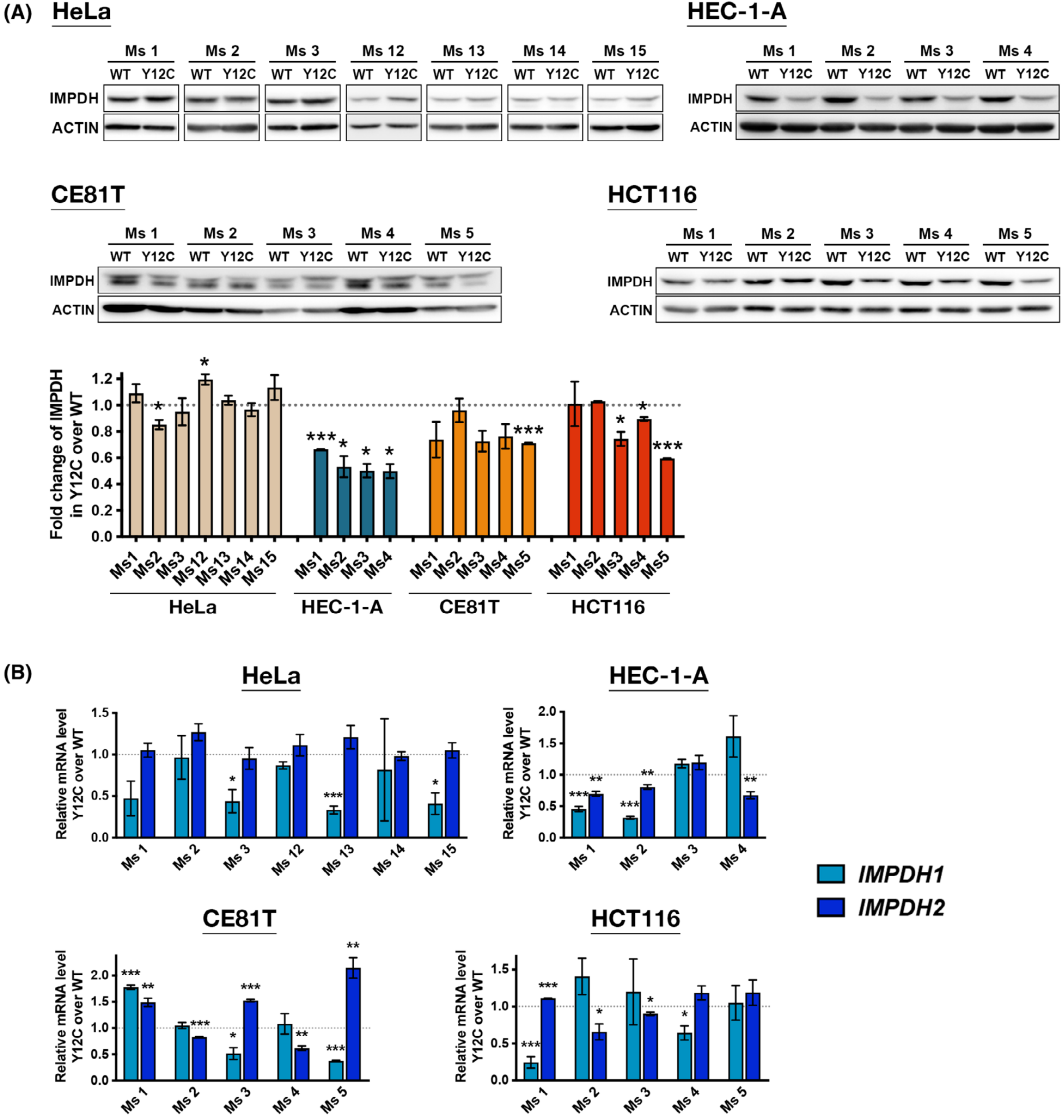
IMPDH2 Y12C point mutation lowers IMPDH levels in some xenografts. (A) Western blot and quantification of IMPDH (normalised to ACTIN) in tumours derived from HeLa, HEC‐1‐A, CE81T, and HCT116 cells. A significant decrease in IMPDH levels is observed in mutant HEC‐1‐A and HCT116 xenografts. (B) Real‐time PCR analysis of IMPDH1 and IMPDH2 mRNA levels in xenografts. Significant reductions in IMPDH1 or IMPDH2 expression levels are detected in some mutant tumours. Images are representative of *n* = 3 independent experiments performed in triplicate, and quantification data are obtained from the same experiments. Statistical significance is evaluated using Student's *t*‐test, with error bars representing SEM (*P < 0.05, **P < 0.01, ***P < 0.001).

In order to understand how tumour growth was suppressed by the loss of IMPDH polymers, we first examined the expression levels of *IMPDH* in four cell lines and their xenografts (Figs 5 and 6). Previous studies have demonstrated that the cytoophidium structure may protect its component proteins from degradation [9, 25]. Similarly, we observed a significant decline in IMPDH protein levels, but not mRNA levels, in HeLa and HCT116 cells by about 20% and 50%, respectively (Fig. 5A,B). Notably, the anti‐IMPDH antibody used for immunoblotting has been tested to label both IMPDH isoforms [10].

IMPDH protein levels significantly decreased in only some tumours, particularly in mutant HEC‐1‐A cell‐derived tumours, where total IMPDH levels were reduced by approximately 50% (Fig. 6A). However, *IMPDH* mRNA levels also significantly decreased in many tumours (Fig. 6B). It is unclear whether the decrease in IMPDH protein levels in mutant xenografts results from reduced protein stability or expression. Nevertheless, the minor reduction in IMPDH levels in mutant HeLa cell‐derived tumours suggests that the growth defects are unlikely to be due to insufficient IMPDH proteins.

### Loss of IMPDH polymers downregulates c‐Myc expression, glycolytic pathway, and PPP in xenografts

In some cancers, such as glioblastoma, mTORC1‐activated tumours, colorectal cancer and a subset of small cell lung cancers, elevated *IMPDH* expression is positively correlated with malignancy [26, 27, 28, 29]. In contrast, IMPDH inhibitors can suppress tumour growth, suggesting their pivotal roles in cancer cell metabolism [1, 26, 27, 29, 30]. Recent studies have revealed that elevated GTP levels increase rRNA and tRNA synthesis in glioblastoma [29], and inhibiting GTP synthesis induces nucleolar stress and growth arrest in both cultured cells and tumour grafts [26, 29].

**Fig. 7:**
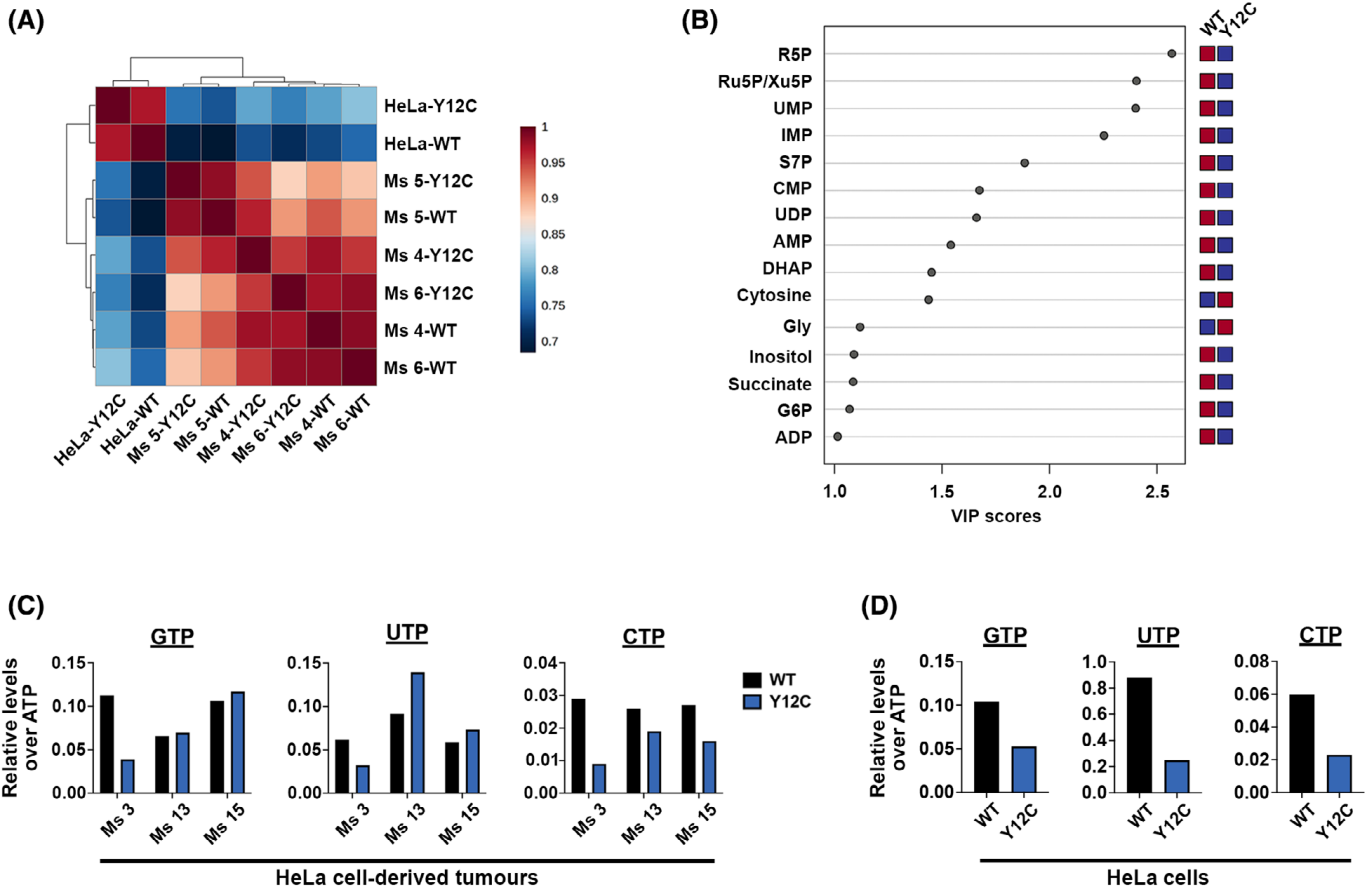
IMPDH2 Y12C point mutation alters the metabolic status of HeLa cell‐derived xenografts. (A) Correlation heatmap of cultured HeLa cells and three pairs of HeLa cell‐derived tumours (Ms 4, 5 and 6). (B) Metabolites discriminating between wild‐type (WT) and mutant xenografts from Ms 4, 5, and 6, with variable importance in the projection (VIP) scores of 1.0 or higher. The bar on the right indicates whether each metabolite is more abundant (red) or less abundant (blue) in each group. (C, D) Nucleotide levels in three pairs of tumours (Ms 3, 13 and 15) derived from HeLa cells (C) and in cultured HeLa cells (D). Levels of GTP, UTP, and CTP are normalised to ATP levels.

To investigate the metabolic pathways affected by the loss of IMPDH polymers and cytoophidia, we performed a metabolomic analysis on HeLa cell lines and tumour pairs from three mice (Ms 4, Ms 5 and Ms 6). The global metabolome of cultured HeLa cells differed significantly from that of the tumours, implying distinct metabolic profiles under different growing conditions (Fig. 7A).

In the tumour samples, intermediates of the PPP pathway (Ru5P, R5P and S7P), an intermediate of the glycolytic pathway (DHAP), and the common precursor of both pathways (G6P) are significantly lower in mutant tumours (Fig. 7B). It is known that both PPP and glycolysis are upstream pathways of nucleotide synthesis and play critical roles in cancer metabolism. R5P can be utilised by PRPS to produce PRPP and is essential for purine and pyrimidine *de novo* synthesis as well as the purine salvage pathway. The low level of R5P may contribute to the decrease of nucleotides, including UMP, AMP and IMP, in mutant tumours (Fig. 7A,B).

Unfortunately, guanine nucleotides were undetectable in these samples, possibly due to levels being below our measurement threshold. Thus, we performed an additional LC–MS/MS analysis to measure nucleotide levels in three other tumour pairs (Ms 3, Ms 13 and Ms 15) and cultured HeLa cells to see if GTP levels are attenuated in IMPDH2 mutant tissues and cells. Using ATP levels as an internal reference, we minimised the influence of potential sample mass loss during preparation. Interestingly, only one mutant tumour (Ms 3) showed lower GTP levels, while all three mutant tumours exhibited relatively lower CTP levels (Fig. 7C). In HeLa cells, however, all three nucleotides were lower in mutant cells (Fig. 7D). Although IMPDH cytoophidia are not commonly seen in HeLa cells under normal culture conditions, it is still possible that a smaller scale of IMPDH polymerisation is required to maintain nucleotide homeostasis. These results suggest that although the IMPDH2 Y12C mutation may disturb GTP synthesis in cultured HeLa cells as anticipated, GTP production may not be the direct cause of tumour growth suppression.

**Fig. 8:**
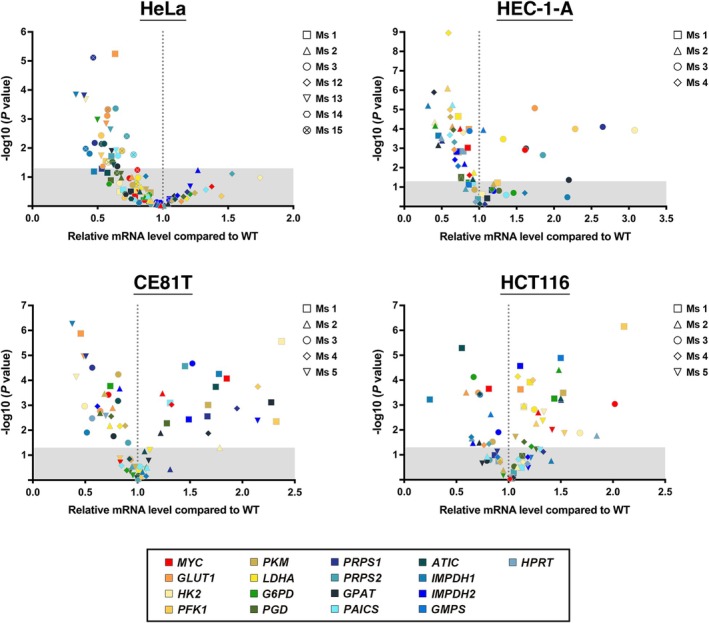
IMPDH2 Y12C point mutation alters metabolic flux in most tumours derived from HeLa, HEC‐1‐A and CE81T cells. Scatter plots showing the relative mRNA levels of selected genes involved in glycolysis, pentose phosphate pathway (PPP) and purine synthesis in mutant HeLa, HEC‐1‐A, CE81T, and HCT116 xenografts compared to their wild‐type (WT) counterparts. Data are derived from *n* = 3 independent experiments performed in triplicate. Genes are colour‐coded, with symbol shapes representing individual tumour samples. Grey areas indicate differences that are not statistically significant (Student's *t*‐test, *P* > 0.05).

We then wondered if the reduction of these metabolites is a consequence of downregulated expression of enzymes in PPP and glycolysis. Real‐time PCR was performed to determine the expression levels of key genes in both pathways (Fig. 8). Notably, due to the small size of the tumours, we could not assess gene expression and metabolome in those with the most severe growth defects. As expected, most genes examined were downregulated in most HeLa, HEC‐1‐A, and CE81T mutant tumours (Fig. 8). In contrast, no obvious expression shift was seen in HCT116 tumours.

It is well known that the oncogene *MYC* is a master transcriptional regulator of several metabolic pathways, including glycolysis and PPP [31]. Many key genes in these pathways are directly controlled by *MYC*. Thus, we measured the expression level of *MYC* in these tumour grafts. In mutant HEC‐1‐A cell‐derived tumours with generally decreased expression levels of the glycolytic pathway and PPP, *MYC* mRNA levels were also slightly reduced in comparison with their wild‐type counterparts (Fig. 8). However, *MYC* expression was not significantly reduced in HeLa and CE81T cell‐derived tumours, even in those with downregulated glycolysis and PPP. These findings suggest that the function of IMPDH has the capacity for reverse regulation, influencing its upstream pathways.

### IMPDH cytoophidia are observed in human cervical cancers

**Fig. 9:**
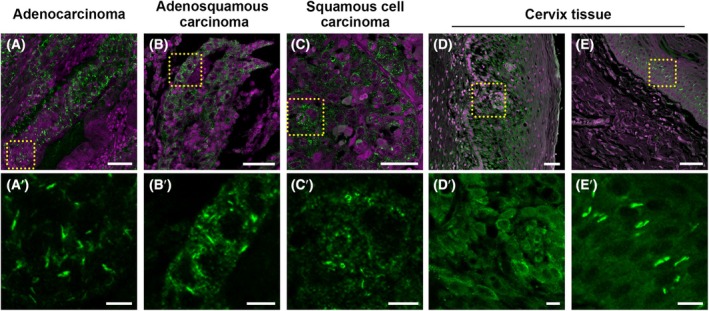
IMPDH cytoophidia are observed in various cervical cancers. (A–E) Immunofluorescence for IMPDH (green) in three types of cervical cancers and non‐cancerous cervix tissues. Yellow dashed boxes indicate selected regions for magnified images (A'–E'). DAPI is shown in magenta. Scale bars are 50 μm in the original images and 10 μm in the magnified images. Many IMPDH cytoophidia are found in cancerous cells and in epithelial cells (mostly parabasal cells) of non‐cancerous cervix tissues. For each tissue type, all tissue samples are analysed, with three images taken per sample; one representative image is presented.

Our data demonstrate that HeLa cells display many IMPDH cytoophidia in tumour grafts, but not under normal culture conditions. In addition, disruption of IMPDH polymers by a single point mutation Y12C in IMPDH2 is sufficient to alter the metabolic status of tumours and reduce tumour growth. In order to further assess the clinical relevance of the IMPDH cytoophidium, we performed immunofluorescence on a human cervical cancer tissue array, which comprises 80 tumour sections and 20 non‐cancerous controls. In most cervical cancer sections, we observed many cytoophidia throughout the tissues (Fig. 9A–C). We categorised the samples into two groups, negative/low and high in cytoophidia, with a cut‐off of 20% of areas showing abundant cytoophidia. Approximately 48% (38 out of 80) of the cervical cancer samples were classified as high in cytoophidia, whereas all non‐cancerous sections were negative/low in cytoophidia.

In all cervical cancer samples, including adenocarcinoma, adenosquamous carcinoma, and squamous cell carcinoma, the abundance of IMPDH cytoophidia is not correlated with patient age, cancer stage, tumour size or lymph node metastasis. However, nearly 70% of cancer tissues with a tumour grade of 3 or higher had cytoophidia, while only around 30% of samples graded 2 or lower showed cytoophidia (Table 2). In non‐cancerous sections, IMPDH cytoophidia could only be found in endothelial cells (Fig. 9D,E). These results suggest that IMPDH formation is a distinctive characteristic of cancerous cells in cervical tissues, consistent with findings in HeLa cell‐derived tumours.

**Table 2:** Correlation between IMPDH cytoophidium expression and clinicopathological characteristics of cervical cancer patients. AT, cancer adjacent cervix tissue; NAT, adjacent normal cervix tissue; Normal, cervix tissue. (*p < 0.05).

	Total	Cytoophidium		*p* chi‐square
		Negative/Low	High	
Pathology diagnosis				
AT/NAT/Normal	20	20 (100%)	0 (0%)	*0.00022
Adenocarcinoma	30	20 (67%)	10 (33%)	
Adenosquamous carcinoma	10	4 (40%)	6 (60%)	
Squamous cell carcinoma	40	18 (45%)	22 (55%)	
Age				
20–29	10	4 (40%)	6 (60%)	0.11130
30–39	16	6 (38%)	10 (63%)	
40–49	24	15 (63%)	9 (38%)	
50–59	18	11 (61%)	7 (39%)	
60–69	6	1 (17%)	5 (83%)	
70–79	6	5 (83%)	1 (17%)	
Stage				
I	58	28 (48%)	30 (52%)	0.16323
II	14	10 (71%)	4 (29%)	
III	6	4 (67%)	2 (33%)	
IV	2	0 (0%)	2 (100%)	
Grade				
1/1–2	16	11 (69%)	5 (31%)	0.17591
2/2–3	42	23 (55%)	19 (45%)	
3	12	4 (33%)	8 (67%)	
Tumour size				
T1	62	30 (48%)	32 (52%)	0.09157
T2	14	10 (71%)	4 (29%)	
T3	2	2 (100%)	0 (0%)	
T4	2	0 (0%)	2 (100%)	
Lymph node metastasis				
N0	76	40 (53%)	36 (47%)	0.91818
N1	4	2 (50%)	2 (50%)	

## Discussion

IMPDH cytoophidia were first identified as abnormal protein aggregates when bound by specific inhibitors [8, 11]. A few years later, it was found that IMPDH cytoophidium formation does not necessarily need to be induced by compounds that directly bind IMPDH. Inhibiting certain metabolic pathways or depriving cells of glutamine can also induce IMPDH cytoophidia in mammalian cell lines [22, 32]. In 2015, we demonstrated that the assembly of IMPDH cytoophidium may actually reflect the upregulation of GTP synthesis in the cell and proposed that IMPDH cytoophidium formation boosts GTP production when cellular demand increases. This idea is further supported by findings showing cytoophidium assembly can be triggered by cell signalling activation, such as T lymphocyte activation and light stimulation in retinal photoreceptor cells [13, 14, 16]. Recent studies have revealed the structural basis of the cytoophidium subunit, the IMPDH polymers, showing that polymerisation increases its tolerance to GTP inhibition. Yet, whether cytoophidium assembly has a functional association with metabolic regulation in the cell is still obscure.

Given the accumulating evidence, we speculate that IMPDH polymerisation and cytoophidium formation might be a mechanism to couple GTP production with active metabolism in certain cell types such as cancer cells. In order to test this idea, we developed a strategy to disrupt IMPDH polymerisation by introducing a Y12C point mutation into endogenous IMPDH1 and IMPDH2 sequences using the ABEmax base editor. The high efficiency and safety of the ABEmax base editor have been comprehensively examined [20]. Not surprisingly, this molecular tool effectively disrupted cytoophidium assembly in all tested cell lines.

Under normal culture conditions, we did not observe significant growth defects in IMPDH2^Y12C^ mutant cells, suggesting that this point mutation does not cause cytotoxicity. Similar results have shown that long‐term overexpression of IMPDH1^Y12C^ protein in HEp‐2 cells neither delays cell growth nor increases cell death [19]. In our parallel study, we successfully introduced the IMPDH2 Y12C mutation to mouse embryonic stem cells (mESCs) and established stable IMPDH2^Y12C^ mESC lines. This mutation completely eliminates IMPDH cytoophidia in mESCs without impairing cell growth or pluripotency under culture conditions [33]. Inhibition of GTP production using IMPDH inhibitors typically leads to cell cycle arrest or apoptosis in highly proliferative cells [30]. However, these effects are not observed in homozygous IMPDH2^Y12C^ mESCs, even though IMPDH2 is the predominant isoform in mESCs [33]. These studies support our hypothesis that the Y12C mutation, similar to the Y12A mutation, only interferes with IMPDH polymerisation but does not directly disrupt enzymatic activity. Thus, this ABEmax‐based genome‐editing strategy provides a promising tool for investigating the functions of IMPDH polymers/cytoophidium in specific cell types or physiological events.

*De novo* GTP synthesis plays pivotal roles in the metabolism of many cancers [30, 34]. According to the human protein atlas database, higher expression levels of IMPDHs also correlate with malignancy in several cancers. Higher IMPDH1 expression is linked to poorer prognosis in renal cancer, liver cancer, urothelial cancer, glioma, and cervical cancer, while high IMPDH2 levels associate with poor prognosis in liver cancer. Since the formation of IMPDH cytoophidium has been proposed as an indicator for active GTP synthesis and the presence of a high amount of IMPDH polymers, it is reasonable to suspect that cancer cells are more likely to have cytoophidia [9, 10]. Indeed, the abundance of IMPDH cytoophidia has been demonstrated as a promising biomarker for acral melanomas [15]. In a cohort of adult diffuse gliomas, nuclear IMPDH cytoophidia were found in 71.1% of IDH mutant lower‐grade gliomas and 13.7% of IDH wild‐type glioblastomas [35]. Herein, we find IMPDH cytoophidia in tissue samples from more than a dozen cancer types. Considering that each tumour section is only 1 mm in diameter, the assembly of IMPDH cytoophidia is likely more common in cancerous tissues than our observations suggest.

By introducing the Y12C mutation to endogenous IMPDH2, we assess the importance of IMPDH polymerisation and cytoophidium formation in multiple cancer cell lines both *in vitro* and *in vivo*. While the IMPDH2 mutation generally leads to downregulation of glycolytic pathway and PPP enzyme mRNA levels in most mutant HEC‐1‐A, CE81T, and HeLa cell‐derived tumours, this trend is less apparent in mutant HCT116 cell‐derived tumours. In addition, despite a reduction in IMPDH protein in all HEC‐1‐A cells‐derived mutant tumours, only a small subset of tumours derived from mutant HeLa, CE81T, and HCT116 cells display a similar decrease. This variability may be attributed to the distinct characteristics and plasticity of individual cancer types or cell lines. In our recent study, a similar shift in gene expression profiles was also observed in cultured mESCs, and the elevation of GTP levels through additional guanosine supplementation restored the expression of these genes [33].

Our previous findings suggest that IMPDH cytoophidium assembly could be promoted by active glycolysis [18]. These observations reinforce the correlation between IMPDH polymerisation and cancer metabolic reprogramming. Interestingly, our results suggest a reverse feedback loop. Most IMPDH2^Y12C^ HEC‐1‐A, CE81T, and HeLa cell xenografts displayed global downregulation of the glycolytic pathway and PPP. Although the mutation results in a 50% decrease in GTP levels in cultured HeLa cells, this trend is not detected in all HeLa cell xenografts. This difference between cultured cells and xenografts could possibly be attributed to higher nucleotide levels in cells cultured in rich medium, as nutrient availability may promote nucleotide accumulation [36]. Higher intracellular GTP levels are expected to suppress IMPDH activity more, resulting in the need for IMPDH polymerisation to maintain GTP synthesis. Therefore, the metabolic reprogramming observed in mutant tumours does not necessarily result from reduced intracellular GTP concentration. Alternatively, it is also possible that the IMPDH2 Y12C mutation indirectly influences other metabolic pathways, such as CTP synthesis, or other functions of IMPDH. IMPDH has been shown to enhance cancer cell growth and invasion through direct interaction with certain proteins, such as B7‐H3, YB‐1 and RAC1 [37, 38, 39]. Meanwhile, we cannot exclude the possibility of unexpected effects from the cysteine substitution on the protein surface, especially in the complex *in vivo* environment. Future studies are required to elucidate the underlying mechanisms of the metabolic alterations and why certain cell types are more sensitive to the IMPDH2 Y12C mutation.

In this study, we aim to extend our current understanding of IMPDH polymerisation from *in vitro* studies to the broader context of cell metabolism. We show that IMPDH cytoophidium formation is a common phenomenon in human tumours. The disruption of IMPDH polymerisation and cytoophidium assembly may alter the metabolic status of certain xenografts, even delaying tumour growth. The successful alteration of cell metabolism in our models by base‐editing provides a basis for future *in vivo* studies on the functions of IMPDH polymerisation and cytoophidium assembly in cancers and normal tissues.

## Materials and Methods

### Specimens

Multiple organ tumour tissue array (MC2082c) and multiple cervix cancer with cervix tissue array (CR1001b) were purchased from US Biomax (now TissueArray.com, Derwood, MD, USA).

### Cell culture

The human embryonic kidney (HEK) 293T cells (RRID:CVCL_0063), cervical cancer cell line HeLa (RRID:CVCL_0030), oesophageal cancer cell line CE81T (RRID:CVCL_Y011) and colon cancer cell line HCT116 (RRID:CVCL_0291) were cultured in Dulbecco's modified Eagle's medium (DMEM) with high glucose and pyruvate (11995065, Gibco, Waltham, MA, USA). Human endometrial cancer cell line HEC‐1‐A (RRID:CVCL_0293) was kept in McCoy's 5A (Modified) medium (16600082, Gibco). Human bladder cancer cell line T24 (RRID:CVCL_0554) and testicular cancer cell line NCCIT (RRID:CVCL_1451) were grown in RPMI‐1640 medium (11875093, Gibco). All media were supplemented with 10% fetal bovine serum (FBS, TMS‐013‐BKR, Millipore, Burlington, MA, USA) and 1% Penicillin–Streptomycin (15140‐122, Gibco). All cell lines were obtained from the National Health Research Institutes Cell Bank, Taiwan, and authenticated by short tandem repeat (STR) profiling before experimentation. Cells were cultured in a 37 °C humid incubator with 5% CO_2_. Mycophenolic acid (MPA) solubilised in DMSO was used in the experiments. All experiments were conducted using mycoplasma‐free cells.

### Establishment and characterisation of mutant cell lines by ABEmax base‐editing

The cancer cell lines were generated using TurboFect™ Transfection Reagent (R0532, Thermo Scientific, Waltham, MA, USA) following the manufacturer's instructions by transfecting plasmids encoding ABEmax‐T2A‐mCherry (modified from pCMV_ABEmax, #112095, Addgene, Watertown, MA, USA) and sgRNA with a puromycin resistance gene (pGL3‐U6‐sgRNA‐PGK‐puromycin, #51133, Addgene) [20, 40]. Two days after transfection, cells were selected with 2 μg·mL^−1^ puromycin. Puromycin‐resistant cells were seeded into individual wells of a 96‐well plate. Single‐cell‐derived colonies were subsequently harvested and sequenced by Sanger sequencing for the targeted DNA region. Colonies confirmed as mutant cells were then pooled to establish the mutant cell line.

### EdU labelling

Cells were incubated with 20 μm of EdU for 15–20 min before fixation with 4% paraformaldehyde. Following fixation, a Click‐iT® azide‐based reaction was carried out as per the manufacturer's protocol (C10340, Invitrogen, Waltham, MA, USA) to attach the Alexa Fluor 647 molecule to the EdU that was incorporated into the newly synthesised DNA.

### Colony formation assay

Different cell lines were seeded in 6‐well plates at varying densities: HeLa cells at 300 cells per well, HCT116 and NCCIT cells at 500 cells per well, CE81T and HEC‐1‐A cells at 1000 cells per well, and T24 cells at 200 cells per well. After 10 days of incubation, colonies were washed with PBS, fixed in 70% (v/v) ethanol for 10 min, and then stained with Giemsa stain (GS500, Sigma‐Aldrich, Burlington, MA, USA). The number of colonies in each well was manually counted.

### Cell cycle analysis

The cells were collected and fixed in ice‐cold 70% (v/v) ethanol for 24 h at 4 °C, then stained with a combination of RNase A (10 μg·mL^−1^, Geneaid, New Taipei City, Taiwan) and propidium iodide (PI, 50 μg·mL^−1^, P4170, Sigma‐Aldrich) in PBS containing 0.5% triton X‐100 for 30 min in the dark. Beckman Coulter FC500 Flow Cytometer was employed to perform the cell cycle analysis.

### Animal

NOD.CB17‐Prkdc^scid^/NCrCrlBltw mice were obtained from BioLASCO Taiwan Co., Ltd. (Taipei City, Taiwan) and housed in a specific pathogen‐free (SPF) facility under a 12‐h light/dark cycle with controlled temperature (20–24 °C) and humidity (40–60%). They were provided with autoclaved food and water with *ad libitum* access. All animal maintenance, handling, and procedures were approved by the Institutional Animal Care and Use Committee of National Taiwan University (protocol number NTU‐107‐EL‐216).

### Xenograft tumour growth assay

After dissociation using a 0.25% trypsin–EDTA solution (25200056, Gibco), 1 × 10^6^ cells were concentrated in 50 μL of culture medium and injected into the thigh muscles of the hind limb of 10‐week‐old immune‐deficient mice using a 26 G × ½″ hypodermic needle. Transplants were collected 5 weeks post‐transplantation, frozen in liquid nitrogen and preserved at −80 °C.

### Cryosection

Tissues were collected and immediately embedded with O.C.T. compound (4583, Tissue‐Tek, Torrance, CA, USA) and stored at −80 °C until use. Tissue sections were obtained using a Leica CM1950 cryostat and mounted onto glass slides coated with silane (5116, Muto Pure Chemicals, Tokyo, Japan).

### Immunofluorescence

Before incubating the tissue sections with antibodies, a series of procedures were conducted on formalin/paraformaldehyde‐fixed and paraffin‐embedded samples. First, the samples were deparaffinised and rehydrated through a sequential incubation process using the following solutions: xylenes for 20 min (2 × 10 min), 100% ethanol for 5 min, 95% ethanol for 5 min, 70% ethanol for 5 min, 50% ethanol for 5 min, and finally H_2_O for 5 min. Subsequently, heat‐induced epitope retrieval was performed at 95 °C for 20 min using Antigen Retrieval AR‐10 (Tris) pH‐10 (HK057‐5K‐GP, BioGenex, Fremont, CA, USA). After allowing the slides to cool down to room temperature, the sections were washed with H_2_O for 5 min. To prevent nonspecific antibody binding, the sections were blocked with Background Sniper blocking reagent (BS966, Biocare Medical, Pacheco, CA, USA) for 15 min at room temperature. All samples, including formalin/paraformaldehyde‐fixed and paraffin‐embedded tissue sections, cryosections, and fixed cells, were incubated with the primary antibody in a solution of 2.5% bovine serum albumin (BSA, A9647, Sigma‐Aldrich) and 0.25% Triton‐X100 in PBS for a minimum of 2 h at room temperature. Following this, the samples were washed with PBS and incubated with a secondary antibody for a minimum of 2 h. After the secondary antibody reaction, samples were stained with DAPI (D9542, Sigma‐Aldrich) to label nuclei. Then, they were washed with PBS and mounted with PBS for imaging. All tissue staining was performed in parallel with control sections stained with only secondary antibodies. Antibodies used in this study include rabbit anti‐IMPDH2 polyclonal antibody (12948‐1‐AP, ProteinTech, Rosemont, IL, USA), Alexa Fluor 488‐conjugated goat anti‐rabbit IgG polyclonal antibody (A11034, Invitrogen) and Alexa 647‐conjugated goat anti‐mouse IgG polyclonal antibody (A21235, Invitrogen). All antibodies were applied at a 1 : 500 dilution. Images were acquired using a laser‐scanning confocal microscope (Leica TCS SP5 II confocal microscope).

### Immunoblotting

Tissue samples were homogenised with a micropestle, and tissue lysates as well as cell lysates were obtained by incubating the samples with RIPA lysis buffer (20‐188, Millipore). The protein content was determined using a Bio‐Rad Protein Assay Kit (5000002, Bio‐Rad, Hercules, CA, USA). The lysates were then loaded onto a 12% polyacrylamide gel and transferred onto a PVDF membrane (GE Healthcare, Chicaco, IL, USA) for protein transfer. Primary and secondary antibodies were diluted in PBST with 5% skimmed milk and incubated overnight for immunolabelling. The antibody labelling was detected using SuperSignal™ West Pico PLUS Chemiluminescent Substrate (34579, Thermo Scientific) and visualised using a chemiluminescence imaging system (GeneGnome XRQ, Syngene, Bangalore, Karnataka, India). The following antibodies were used: rabbit anti‐IMPDH2 polyclonal antibody (1 : 10 000, 12948‐1‐AP, ProteinTech), rabbit anti‐HPRT polyclonal antibody (1 : 5000, GTX113466, GeneTex, Irvine, CA, USA), HRP‐conjugated mouse anti‐β‐ACTIN monoclonal antibody (1 : 3000, HRP‐60008, ProteinTech) and HRP‐conjugated goat anti‐rabbit IgG polyclonal antibodies (1 : 10 000, 31460, Invitrogen).

### Real‐time qPCR

Total RNA was extracted with the GENEzol™ TriRNA Pure Kit (GZX100, Geneaid) following the manufacturer's instructions. The cDNA solution was prepared using GoScript™ Reverse Transcriptase (A2801, Promega, Madison, WI, USA). For real‐time PCR analysis, KAPA SYBR® FAST qPCR Master Mix (KK4609, Roche, Basel, Switzerland) was used according to the manufacturer's protocol. Real‐time qPCR was conducted on a Bio‐Rad CFX384 qPCR System with 40 cycles of 3 s at 95 °C and 20 s at 60 °C, followed by a thermal denaturing step to generate dissociation curves to verify amplification specificity. All genes were normalised using the CT value of *ACTIN*. The primers used are shown in Table 3.

**Table 3:** List of primers used for qPCR.

Human	Forward primer (5′–3′)	Reverse primer (5′–3′)
*ACTIN*	GAGAAAATCTGGCACCACACC	GGATAGCACAGCCTGGATAGCAA
*ATIC*	TGCGACGAACTGGGAATCAT	AGGCGTGACTGTTCACCTAC
*G6PD*	TACACTTCGGGGCTGCGAG	AGCCCACGATGAAGGTGTTT
*GLUT1*	TCTGGCATCAACGCTGTCTTC	CGATACCGGAGCCAATGGT
*GMPS*	AGGAACAAGGATTCCGTGCTA	GAAGGCCCCTGAATAATGAACA
*GPAT*	GATGTAAGCACACAAGTGAGGA	TCCGACTCATTAGGCTTTCTTTC
*HK2*	GAGCCACCACTCACCCTACT	CCAGGCATTCGGCAATGTG
*HPRT*	AGGCGAACCTCTCGGCTTTC	CTAATCACGACGCCAGGGCT
*IMPDH1*	TTCGTGCCCTACCTCATAGC	ATGGACCGAAGGACAGACAG
*IMPDH2*	AGTGGCTCCATCTGCATTACG	ACCTTGTACACTGCTGTTGCTTG
*LDHA*	ATGGCAACTCTAAAGGATCAGC	CCAACCCCAACAACTGTAATCT
*MYC*	GTCAAGAGGCGAACACACAAC	TTGGACGGACAGGATGTATGC
*PAICS*	TTGCAGAAGAATAGCAACTGGTT	CACTGTGGGTCATTATTGGCAT
*PFK1*	GGTGCCCGTGTCTTCTTTGT	AAGCATCATCGAAACGCTCTC
*PGD*	GACATCATCATTGACGGAGGAAA	GGGCCACGCTTCTTTGTTC
*PKM*	ATGTCGAAGCCCCATAGTGAA	TGGGTGGTGAATCAATGTCCA
*PRPS1*	CCAGGAGACCTGAGTGACCT	GCAGGACCGGAGAAGATTCC
*PRPS2*	CTGGGGCGGATCACATCATC	CCGCATACAAATTATCCACAGGA

### Metabolomic analysis using mass spectrometry

Metabolites were extracted from 10 mg of tissue samples and 10^6–7^ cells using 600 μL and 200 μL of extraction liquid (a mixture of methanol, acetonitrile and water in a 2 : 2 : 1 ratio with 2 ppm of internal standard), respectively. Tissue samples were homogenised using a bead beater, and the supernatant was transferred to a fresh tube. The extracts were then dried by a vacuum concentrator. The samples were analysed using an Agilent 1290 Infinity II ultra‐high performance liquid chromatography (UHPLC) system coupled online to an Agilent 6545XT quadrupole time‐of‐flight (Q‐TOF) mass spectrometer with Dual Agilent Jet Stream (AJS) electrospray ionisation (ESI) source, operated in positive and negative full‐scan mode, with collection from m/z of 60–1500. Separation was achieved using an ACQUITY UPLC BEH amide column (1.7 μm, 2.1 × 100 mm, Waters Corp., Milford, MA, USA), with mobile phases composed of H_2_O (eluent A) and 90% acetonitrile (eluent B), both eluents with 15 mm ammonium acetate and 0.3% NH_4_OH, at a flow rate of 300 μL·min^−1^ and an injection volume of 2 μL. The acquired chromatograms and mass spectral peaks were processed using agilent qualitative analysis 10.0 and agilent profinder 10.0 software (Agilent, Santa Clara, CA, USA). The concentrations of each detected metabolite were normalised to the internal standard (fenclonine) and log10‐transformed before analysis using metaboanalyst 5.0 online software. Supervised partial least‐squares discriminant analysis and variable importance in projection analysis were performed to identify a set of metabolites that discriminated between wild‐type and mutant groups. The results of hierarchical clustering were presented as a heat map.

### Nucleotide analysis

The determination of ATP, UTP, CTP and GTP was performed using a Waters UHPLC coupled to a Waters Xevo TQS (Waters, Manchester, UK). In brief, 10^7^ cells or tumour tissues were homogenised and lysed in 80% methanol. After centrifugation at 13 000 ***g*** for 10 min, the supernatants were collected and dried. Pellets were resuspended in water and analysed. The chromatography was carried out on an Atlantis Premier BEH Z‐Hilic column (1.7 μm, 2.1 × 100 mm, Waters, Milford, CT, USA) and a gradient of water with 15 mm ammonium bicarbonate (pH 9.0) and 90% acetonitrile with 15 mm ammonium bicarbonate (pH 9.0) at a flow rate of 0.5 mL·min^−1^. The gradient program was as follows: 0–5 min, 90–65% B; 5–6 min, 65% B; 6–6.5 min, 65–90% B and keep 90% B for 3 min. The column was set at 30 °C. The triple quadrupole mass spectrometry was operated in negative electrospray ionisation (ESI) mode and multiple reaction monitoring (MRM) scan type. Specific parameters of compounds and source parameters were optimised through direct infusion. The MS ion source parameters were set as follows: gas flow was 1000 L·h^−1^ at 500 °C; nebuliser pressure was 7 bar; source temperature was 150 °C; capillary voltage 1500 V. MassLynx software v4.2 (Waters) and TargetLynx (Waters) were used for data acquisition and analysis, respectively.

### Statistical analysis

Statistical analysis was performed using graphpad prism (Boston, MA, USA) software with Student's *t*‐test, and error bars in all graphs represent the standard error of the mean (SEM). For cytoophidium‐ and EdU‐positive cell quantification, images were analysed using fiji (Bethesda, MD, USA) software. Data were obtained from at least three independent experiments, with each quantification involving the examination of over 100 cells. Western blot experiments were performed at least three times with independent samples, and band intensities were measured and normalised to a housekeeping reference using fiji software. Cancer tissue samples with less than 20% of the area displaying cytoophidia were classified as negative or low for cytoophidium. The correlation between cytoophidium scores and clinicopathological characteristics was evaluated using chi‐square analysis.

## Conflict of interest

The authors declare no conflict of interest.

## Author contributions

C‐CC, MP and GDK conceived and designed the studies. C‐CC, MP, GDK, L‐KT, ZZ and L‐MP performed the experiments and analysed the data. L‐YS and J‐LL supervised the project. C‐CC, MP, L‐YS and J‐LL wrote and edited the manuscript with inputs from GDK.

## Peer review

The peer review history for this article is available at https://www.webofscience.com/api/gateway/wos/peer‐review/10.1111/febs.70086.

## Data Availability

The data that support the findings of this study are available from the corresponding author at liujl3@shanghaitech.edu.cn or liyingsung@ntu.edu.tw upon reasonable request.
